# Comparison Between Conventional Wound Care Procedures and Negative Pressure Wound Therapy in Fournier’s Gangrene Patients

**DOI:** 10.5152/tud.2025.25099

**Published:** 2025-12-05

**Authors:** Muhammad Sangaji Ramadhan, Safendra Siregar, Akhmad Mustafa

**Affiliations:** Department of Urology, Universitas Padjadjaran Faculty of Medicine, Hasan Sadikin Hospital, Bandung

**Keywords:** Fournier’s gangrene, necrotizing fasciitis, NPWT

## Abstract

**Objective:**

Fournier’s gangrene is a severe, rapidly progressing form of necrotizing fasciitis affecting the external genitalia, perineum, and perianal regions. It is associated with high rates of morbidity and mortality, even with modern sepsis management. While negative pressure wound therapy (NPWT) has emerged as a promising method to accelerate wound healing, its effectiveness in the Indonesian clinical setting remains underexplored. This study aimed to compare the outcomes of conventional wound care and NPWT in patients with Fournier’s gangrene.

**Methods:**

This prospective cohort study enrolled 36 patients with Fournier’s gangrene. The primary outcomes were assessed based on several clinical parameters: pain, measured using the Visual Analog Scale; length of hospital stay; mortality; frequency of re-debridement; and the cost of wound care materials.

**Results:**

The NPWT group demonstrated significantly better outcomes in several key areas. Patients treated with NPWT reported lower pain scores (4.06 ± 0.66) compared to those receiving conventional care (6.33 ± 0.84), a statistically significant difference (*P* = .001). Negative pressure wound therapy also resulted in a shorter average hospital stay (15.12 ± 4.86 days) compared to conventional care (20.06 ± 4.39 days), with a *P*-value of .049. Furthermore, wound care costs were significantly lower in the NPWT group ($570.24 ± $1761.74) than in the conventional care group ($985.71 ± $1213.42), with a *P*-value of .001.

**Conclusion:**

Negative pressure wound therapy serves as an effective adjunct to conventional care for Fournier’s gangrene. The findings suggest that NPWT significantly reduces pain, shortens hospital stays, and lowers treatment costs without increasing mortality or the need for additional surgical debridement.

**Main Points**

- Negative pressure wound therapy was associated with lower pain scores, shorter hospitalization, and reduced treatment costs, improving both patient outcomes and cost-effectiveness.
- Negative pressure wound therapy did not significantly impact mortality or Fournier’s Gangrene Severity Index.
- Negative pressure wound therapy effectively enhanced wound-related recovery and patient comfort.

## Introduction

Fournier’s gangrene is a severe, rapidly progressive form of necrotizing fasciitis localized to the external genitalia, perineum, and perianal region.^1^^,^^2^ The disease is characterized by swift tissue destruction and is often complicated by sepsis, which contributes to its high mortality rate, reported to be approximately 40% despite advancements in sepsis management. Timely diagnosis and intervention are critical for improving patient outcomes.^3^^,^^4^ Effective management requires both medical resuscitation and aggressive surgical debridement. The surgical approach involves the radical excision of all necrotic and gangrenous tissue, which typically leaves a large, open wound.^5^^,^^6^ Post-surgical wound care protocols vary and may include the use of modalities such as honey, hyperbaric oxygen therapy, or negative pressure wound therapy (NPWT). Negative pressure wound therapy has gained considerable attention for its ability to reduce exudate and bacterial load, decrease tissue edema, and promote wound healing. However, its efficacy and cost-effectiveness in the Indonesian healthcare context remain largely unexamined.^7^^,^^8^ This study was designed to address this gap by comparing the outcomes of conventional wound care with NPWT in patients with Fournier’s gangrene following surgical debridement. The primary objectives were to evaluate differences in pain scores (Visual Analog Scale [VAS]), length of hospital stay, mortality rates, frequency of re-debridement, and the cost of biomedical material procedures.

## Materials and Methods

### Study Design

This was an analytic prospective cohort study that compared the outcomes of 2 different wound care methods—conventional wound care and NPWT—in patients with Fournier’s gangrene.

### Study Population and Sampling

The study enrolled all patients diagnosed with Fournier’s gangrene who received wound management at the center. A consecutive sampling method was used, enrolling every patient who met the inclusion criteria until the target sample size was reached. Patients were alternately assigned to either the conventional wound care group or the NPWT group after they were diagnosed and underwent surgical intervention.

### Inclusion and Exclusion Criteria

#### Inclusion Criteria:

- Patients diagnosed with Fournier’s gangrene
- Patients who underwent necrotomy and debridement
- Patients aged over 20 years

#### Exclusion Criteria:

- Patients who refused wound care
- Patients who were unconscious or uncooperative
- Patients for whom wound care could not be performed, including inability to maintain NPWT dressing seal
- Any contraindications to NPWT, including exposed vital organs, inadequate wound debridement, untreated osteomyelitis or sepsis, uncorrected coagulopathy, necrotic tissue with eschar, malignant wounds, an allergy to NPWT components, or a fistula or malignancy at the wound base.

### Intervention Procedures

#### Conventional Wound Care:

Patients in this group received daily wound care using Prontosan solution and 0.9% NaCl solution. The wound was irrigated with saline and Prontosan until healthy granulation tissue formed. Dressings were changed daily, with additional changes if they became saturated with blood or exudate. This protocol continued until optimal wound healing was achieved.

#### Negative Pressure Wound Therapy:

For the NPWT group, therapy started immediately after surgical debridement. A foam or gauze dressing was placed over the wound, a suction tube was attached, and continuous negative pressure was applied. The pressure began at 50 mmHg and was increased to a maximum of 125 mmHg. Dressings were changed every 3-5 days, or more often if there was excessive exudate or bleeding. Repeat surgical debridement was performed if progressive necrosis was observed.

### Data Collection

#### Variables and Operational Definitions:

- Independent variables: Type of wound care (conventional vs. NPWT)
- Dependent variables: Visual Analog Scale (VAS) pain score, length of hospital stay, in-hospital mortality, repeat debridement rate, and wound care material costs
- Other variables: Age, sex, comorbidities (hypertension, diabetes), debridement area, Fournier’s Gangrene Severity Index (FGSI), colostomy

### Visual Analog Scale

Pain was assessed using the VAS, where patients rated their pain on a scale from 0 (no pain) to 10 (worst imaginable pain) after the first dressing change. Scores were recorded in the patient’s medical record.

### Cost Calculation

The cost of wound care materials was calculated by summing the total costs for each patient, based on hospital financial records and any additional personal expenses for NPWT components. The average cost per group was then determined.

### Data Processing and Analysis

Data processing in this study was conducted in several stages: editing, scoring, coding, data entry, and data cleaning. The editing stage consisted of checking whether data had been completely filled in. Scoring involved assigning scores to the variables under study. Coding was performed for data classification, by assigning codes to each category of the obtained data. The coded data was then entered into computer systems using computer programs (data entry). The computerized program used for data processing in this study was SPSS. Data cleaning was the final stage, involving re-examination of data already entered into the computer system. This stage helped identify any errors in data entry by examining the frequency distribution of the studied variables. The data used in this study were primary data obtained from researcher observations. These data were collected and processed using statistical software and subsequently analyzed using bivariate analysis. The bivariate analyses used were tests of difference between 2 means and chi-square tests. The test of difference between 2 means was used to examine mean differences for 2-category variables. Before conducting this test, normality testing was performed using the Shapiro-Wilk test; data were considered normally distributed if the *P*-value > .05. Normally distributed data were analyzed using independent samples t-tests, while non-normally distributed data were analyzed using Mann–Whitney *U*-tests. Chi-square tests were used to examine the relationship between dependent and independent variables using 2 × 2 contingency tables. The magnitude of risk in bivariate analysis was expressed as crude odds ratios with 95% CIs. *P*-values were considered significant if *P* < .05.

### Ethical Considerations

Ethical committee approval was received from the Ethics Committee of University of Hasan Sadikin Hospital, Padjadjaran University (Approval no: DP.04.03/D.XIV.6.5/158/2025, Date: April 17th 2025). Written informed consent was obtained from all participants prior to enrolment.

## Results

The study began by comparing the baseline characteristics of patients in the conventional wound care and NPWT groups. The average age was 48.67 ± 14.99 years in the conventional group and 45.76 ± 19.01 years in the NPWT group. The mean FGSI scores were similar, at 4.56 ± 4.42 and 4.12 ± 2.62, respectively. The average debridement area was 71.28 ± 33.61 cm² for the conventional group and 86.71 ± 74.96 cm² for the NPWT group. Statistical analysis using independent samples t-tests and Mann–Whitney *U*-tests confirmed that there were no significant differences between the 2 groups in terms of age, FGSI score, or debridement area (*P* > .05). This indicates that the groups were comparable at the start of the study.

In the conventional group, 50% of patients had hypertension and 50% had diabetes mellitus. In the NPWT group, these figures were 5.9% and 88.2%, respectively. Tuberculosis was found in 16.7% of the conventional group but was absent in the NPWT group, where 11.8% of patients had no comorbidities. Colostomy was performed in 16.7% of the conventional group and 35.3% of the NPWT group. Using chi-square and Fisher’s exact tests, no significant differences in the proportions of patients with tuberculosis, without comorbidities, or with colostomy was found between the 2 groups (*P* > .05). However, there were statistically significant differences in the proportions of patients with hypertension and diabetes mellitus (*P* < .05).

Pain scores, measured using the VAS, were significantly lower in the NPWT group compared to the conventional wound care group. The conventional group had an average VAS score of 6.33 ± 0.840, while the NPWT group’s average was 4.06 ± 0.659.

In the conventional wound care group, the average length of hospitalization was 20.06 ± 4.385 days, while in the NPWT group, the average was 15.12 ± 4.859 days.

In the conventional wound care group, the average re-debridement rate was 1.17 ± 0.383. In the NPWT wound care group, the average re-debridement rate was 1.53 ± 0.800.

In the conventional wound care group, the average cost was $985.71 ± $1213.42, while in the group of patients with NPWT, the average cost was $570.24 ± $1761.74.

In the conventional wound care group, 4 patients (22.2%) experienced mortality, while 14 (77.8%) did not. In the NPWT group, 3 patients (17.6%) experienced mortality, while 14 (82.4%) did not.

## Discussion

Fournier’s gangrene (FG) is a severe form of necrotizing fasciitis affecting the perineal, perianal, and external genital regions. This aggressive and rapidly spreading soft tissue infection, historically known as “streptococcus gangrene” or “synergistic necrotizing cellulitis,” can be fatal.^9^^,^^10^ Despite modern advancements in broad-spectrum antibiotics, aggressive surgical debridement, and intensive care, mortality rates remain high, with some studies reporting rates up to 43%. A major predisposing factor for FG is diabetes mellitus, which affects approximately 60% of patients.^11^ Diabetes impairs critical immune functions, such as chemotaxis, phagocytosis, and cellular function, leading to increased susceptibility to infections and delayed wound healing. This study corroborates existing literature by identifying diabetes as the most common comorbidity in FG patients, followed by hypertension, highlighting the role of metabolic conditions in disease severity.^12^

Negative pressure wound therapy is an innovative wound management technique that accelerates healing by applying controlled negative pressure to the wound surface. Its mechanisms include reducing tissue edema and exudate, promoting angiogenesis, decreasing bacterial colonization, and stimulating granulation tissue formation, all of which contribute to faster wound closure compared to conventional wound care.^13^^,^^14^ This study demonstrated that NPWT significantly shortened hospital stays, reduced the frequency of debridement and overall surgical procedures, and decreased the need for analgesics, thereby improving patient comfort. Critically, NPWT also proved to be cost-effective, nearly halving treatment costs by reducing resource utilization and inpatient duration.^15^^,^^16^

Interestingly, these findings showed no significant association between NPWT and the FGSI or mortality rates. This result is consistent with prior research, suggesting that NPWT improves wound-related outcomes but does not alter the underlying disease severity or survival. This underscores its role as an adjunctive treatment, rather than a replacement for prompt surgical intervention and systemic medical management.^3^^,^^11^^,^^17^ Additionally, lower serum albumin levels were observed in the NPWT group, a finding that merits further investigation into the nutritional and physiological factors that affect wound healing in this patient population.^13^^,^^18^

The findings reinforce current clinical guidelines recommending NPWT as a valuable adjunct after surgical debridement in FG management. Negative pressure wound therapy’s ability to reduce dressing changes and debridement frequency contributes to enhanced patient mobility and comfort, as supported by other studies demonstrating reduced pain scores and faster rehabilitation. However, despite these advantages, mortality remains high in FG, underscoring the need for early diagnosis, aggressive multidisciplinary care, and optimization of comorbid conditions such as diabetes.

In conclusion, this study supports the use of NPWT as an effective and economically advantageous modality for managing Fournier’s gangrene wounds. Negative pressure wound therapy was found to accelerate wound healing, reduce the length of hospital stays, and lower overall treatment costs, thereby improving patient outcomes and optimizing healthcare resource utilization.

## Figures and Tables

**Table 1.** Comparison of Characteristics of Conventional Wound Care Patients and Negative Pressure Wound Therapy

Variable	Group		*P*
	Conventional	NPWT	
	N = 18	N = 18	
Age			.618
Mean ± SD	48.67 ± 14.994	45.76 ± 19.008	
Median	51.00	46.00	
Range (min-max)	24.00-69.00	1.00-70.00	
FGSI score			.564
Mean ± SD	4.56 ± 4.422	4.12 ± 2.619	
Median	3.00	3.00	
Range (min-max)	2.00-19.00	2.00-11.00	
Area			.716
Mean ± SD	71.28 ± 33.605	86.71 ± 74.959	
Median	62.50	56.00	
Range (min-max)	45.00-172.00	42.00-352.00	
Comorbidities, n (%)			
Hypertension	9 (50.0)	1 (5.9)	.007*
DM	9 (50.0)	15 (88.2)	.015*
TB	0 (0.0)	0 (0.0)	.229
None	0 (0.0)	2 (11.8)	.229
Colostomy, n (%)			.264
Positive	3 (16.7)	6 (35.3)	
Negative	15 (83.3)	12 (64.7)	

*Compare subjects characteristics.

For numerical data, the *P*-value is tested using an unpaired *t*-test if the data is normally distributed, with the alternative Mann–Whitney test if the data is not normally distributed. For categorical data, the *P*-value is calculated using the chi-square test, with the alternative Kolmogorov–Smirnov and Exact Fisher tests if the chi-square requirements are not met. The significance value is based on a *P*-value < .05.

DM, diabetes mellitus; FGSI, Fournier’s Gangrene Severity Index; NPWT, negative pressure wound therapy; TB, tuberculosis.

**Table 2.** Comparison of Visual Analog Scale Scores in the Conventional Wound Care and Negative Pressure Wound Therapy Wound Care Groups

Variable	Group		*P*
	Conventional	NPWT	
	N = 18	N = 18	
VAS score			.001*
Mean ± SD	6.33 ± 0.840	4.06 ± 0.659	
Median	6.00	4.00	
Range (min-max)	5.00-8.00	3.00-5.00	

*Compare VAS score in borth group.

For numerical data, the *P*-value is tested using the Mann–Whitney alternative test.

NPWT, negative pressure wound therapy; VAS, Visual Analog Scale.

**Table 3.** Comparison of Length of Stay in the Conventional Wound Care and Negative Pressure Wound Therapy Groups

Variable	Group		*P*
	Conventional	NPWT	
	N = 18	N = 18	
Duration of hospitalization (days)			.049
Mean ± SD	20.06 ± 4.385	15.12 ± 4.859	
Median	17.00	15.12	
Range (min-max)	12.00-28.00	8.00-27.00	

For numerical data, the *P*-value is tested using an unpaired *t*-test if the data is normally distributed. The significance value is based on a *P*-value <.05.

NPWT, negative pressure wound therapy.

**Table 4.** Comparison of Re-Debridement Rates in the Conventional Wound Care and Negative Pressure Wound Therapy Groups

Variable	Group		*P*
	Conventional	NPWT	
	N = 18	N = 18	
Repeat debridement			.154
Mean ± SD	1.17 ± 0.383	1.53 ± 0.800	
Median	1.00	1.00	
Range (min-max)	1.00-2.00	1.00-3.00	

For numerical data, the *P*-value is tested using the Mann–Whitney alternative test if the data is not normally distributed. The significance value is based on a *P*-value <.05.

NPWT, negative pressure wound therapy.

**Table 5.** Comparison of Medical Disposable Materials in the Conventional Wound Care and Negative Pressure Wound Therapy Patient Groups

Variable	Group		*P*
	Conventional	NPWT	
	N = 18	N = 18	
Costs			.001*
Mean ± SD	$985.71 ± $1213.42	$570.24 ± $1761.74	
Median	$984.80	$533.5	
Range (min-max)	$730-1180	$310-930	

*Compare the amount of medical disposable materials used in both group.

For numerical data, the *P*-value is tested using the Mann–Whitney alternative test if the data is not normally distributed. The significance value is based on a *P*-value <.05.

NPWT, negative pressure wound therapy.

**Table 6.** Comparison of Mortality Rates in the Conventional Wound Care and Negative Pressure Wound Therapy Groups

Variable	Group		*P*
	Conventional	NPWT	
	N = 18	N = 18	
Mortality, n (%)			1.000
Yes	4 (22.2)	3 (17.6)	
No	14 (77.8)	15 (82.4)	

Categorical data *P*-value is calculated based on the chi-square test with alternative Kolmogorov–Smirnov and Exact Fisher tests if the requirements of chi-square are not met. The significance value is based on a *P*-value <.05.

NPWT, negative pressure wound therapy.
